# Nucleic acid degradation after long-term dried blood spot storage

**DOI:** 10.1111/1755-0998.13979

**Published:** 2024-05-23

**Authors:** Juan Li, Gabriela M. Ulloa, Pedro Mayor, Meddly L. Santolalla Robles, Alex D. Greenwood

**Affiliations:** 1 Department of Wildlife Diseases, Leibniz Institute for Zoo and Wildlife Research (IZW), Berlin, Germany; 2 Programa de Pós-Graduação em Saúde e Produção Animal na Amazônia, Universidade Federal Rural da Amazônia (UFRA), Belém, Pará, Brazil; 3 Grupo de Enfermedades Infecciosas Reemergentes, Universidad Científica del Sur (UCSUR), Lima, Peru; 4 ComFauna, Comunidad de Manejo de Fauna Silvestre en la Amazonía y en Latinoamérica, Iquitos, Peru; 5 Departament de Sanitat i Anatomia Animals, Universitat Autònoma de Barcelona, Bellaterra, Spain; 6 Emerge, Emerging Diseases and Climate Change Research Unit, School of Public Health and Administration, Universidad Peruana Cayetano Heredia, Lima, Peru; 7 School of Veterinary Medicine, Freie Universität Berlin, Berlin, Germany

**Keywords:** DBS, hybridization capture, mitochondrial genome, nucleic acid, virus

## Abstract

Collecting and preserving biological samples in the field, particularly in remote areas in tropical forests, prior to laboratory analysis is challenging. Blood samples in many cases are used for nucleic acid-based species determination, genomics or pathogen research. In most cases, maintaining a cold chain is impossible and samples remain at ambient temperature for extended periods of time before controlled storage conditions become available. Dried blood spot (DBS) storage, blood stored on cellulose-based paper, has been widely applied to facilitate sample collection and preservation in the field for decades. However, it is unclear how long-term storage on this substrate affects nucleic acid concentration and integrity. We analysed nucleic acid quality from DBS stored on Whatman filter paper no. 3 and FTA cards for up to 15 years in comparison to cold-chain stored samples using four nucleic acid extraction methods. We examined the ability to identify viral sequences from samples of 12 free-ranging primates in the Amazon forest, using targeted hybridization capture, and determined if mitochondrial genomes could be retrieved. The results suggest that even after extended periods of storage, DBS will be suitable for some genomic applications but may be of limited use for viral pathogen research, particularly RNA viruses.

## 1 | INTRODUCTION

In-field preservation of biological samples is of paramount importance to their subsequent utility in laboratory settings. This is particularly true if the nucleic acids are the intended downstream analysis target. Various preservation methods have been employed to store samples for nucleic acid-based research in the field including freezing where electricity or liquid nitrogen storage is possible, preservation in storage buffers such as RNALater and ethanol (Camacho-Sanchez et al., 2013; Nsubuga et al., 2004; Soto-Calderon et al., 2009), and several types of filter paper (Hearps et al., 2010; Hsiao et al., 1999; Samsonova et al., 2022; Smith & Burgoyne, 2004). Snap freezing maintains the integrity of nucleic acids ideal for downstream genomic applications (da Cunha Santos, 2018; Mareninov et al., 2013; Riesgo et al., 2012; Tang et al., 2012) as it effectively halts enzymatic activity, slows nucleic acid degradation, and minimizes microbial growth (Naber, 1996; Shabihkhani et al., 2014). However, its successful implementation requires overcoming the logistical challenges associated with maintaining low temperatures under various environmental conditions. In many cases, such as remote forest locations requiring weeks of travel, these challenges can be unsurmountable.

Storage buffers represent a good bridge method to preserve nucleic acids at ambient temperatures in the field for several days or weeks before transfer to a laboratory setting where they can be frozen (Camacho-Sanchez et al., 2013). However, preservation buffers preserve nucleic acids less effectively over time than freezing particularly under extreme environments (Camacho-Sanchez et al., 2013; Riesgo et al., 2012), making them more suited for short-term storage. Furthermore, storage buffer is relatively expensive and can be cost-prohibitive for large field-based studies.

Filter paper storage is easy to implement and transport even in remote field sites. The most widely applied sample type is dried blood spots (DBS) (McClendon-Weary et al., 2020; Samsonova et al., 2022). DBS samples were first applied in clinical settings to detect metabolites indicative of phenylketonuria in newborns (Guthrie & Susi, 1963; Pitt, 2010; van Vliet et al., 2020). The use of DBS has subsequently been extended to store samples in various medical and animal research settings (Lehmann et al., 2013; McClendon-Weary et al., 2020). Such studies have targeted proteins (Martin & Cooper, 2014), lipids (Koulman et al., 2014), and nucleic acids (Tuaillon et al., 2020). DBS has been used extensively in medical diagnostics and bio-banking (Capiau et al., 2019; Demirev, 2013; Lim, 2018; McClendon-Weary et al., 2020; Russcher et al., 2020), while its potential significance in the fields of animal biology, particularly for wildlife may be underestimated (Samsonova et al., 2022).

DBS are obtained by spotting a small amount of blood onto filter paper, a stable and compact format for transportation and storage, and allowing it to dry at room temperature (McClendon-Weary et al., 2020). Multiple types of specialized filter paper have been designed to optimize the collection and stability of biological materials of interest for a variety of research purposes, such as Whatman 903 protein saver, Flinders Technology Associates (FTA) Cards, and Nucleic-Card (TM) (Marchand et al., 2021; Molteni et al., 2013; Uttayamakul et al., 2005). FTA cards are the best-established option in nucleic acid-targeted research (Cardona-Ospina et al., 2019; da Cunha Santos, 2018; Hashimoto et al., 2019; Longhi et al., 2023; Rahikainen et al., 2016; Tam et al., 2016). FTA cards rely on a porous matrix of cellulose fibre to trap nucleic acid molecules, while they are additionally impregnated with proprietary chemicals that can denature proteins and reduce microbial growth, preventing enzymatic degradation of nucleic acids. Untreated filter paper was reportedly comparable in preserving viral nucleic acids compared with more expensive chemically-treated FTA cards (Jones et al., 2012), even for prolonged storage (up to 9 months) under tropical environmental conditions (Michaud et al., 2007). However, there is a scarcity of research on nucleic acid preservation on treated and non-treated filter paper over extended periods, particularly as it applies to wildlife and conservation research, fields which are particularly dependent on long-term field storage of collected samples.

DBS on filter paper have been used in a number of animal genetic and pathogen studies. For example, rabies virus in dried brain tissue was stably detected on filter paper for up to 2 years at ambient conditions (Rasolonjatovo et al., 2020; Wacharapluesadee et al., 2003). DNA from avian DBS on FTA cards was preserved for up to 44 months (Smith & Burgoyne, 2004). Fowler et al. (2012) demonstrated that DNA on FTA cards can be used to amplify multiple single-nucleotide polymorphism loci even after 3 years of DBS storage. The aforementioned studies were based on target-amplification approaches that can work with minimal starting DNA amounts and tolerate substantial DNA degradation. However, many samples that are retrieved and stored could benefit from recent advances in genomics methods or may represent the only resources for genomic information on rare species (Andrews et al., 2018; Carroll et al., 2018; Schilling et al., 2022). In such cases, quality assessment in terms of fragment size and concentration of DNA or RNA is required for analysis. For pathogen testing, there is the additional variable of pathogen concentration in a given sample, which may be low in which case degradation may exacerbate general detection-associated problems.

In the current study, we measured DNA integrity and concentration from wildlife collected on two types of filter paper (Whatman filter paper no. 3 (W3) and FTA cards) and stored from 1 to 15 years, compared with immediately cold-chain stored samples. We determined (1) how the quality of DNA and RNA on DBSs changed over time, compared with frozen human whole blood samples; (2) the storage performance of FTA and W3 cards; (3) the impact of nucleic acid extraction method; (4) virus sequence retrieval by target capture and sequencing; and (5) retrieval of non-target nuclear and mitochondrial DNA sequences.

## 2 | MATERIAL S AND METHODS

### 2.1 | Sampling and storage

In the current study, we used 178 (117 FTA and 61 W3) dried blood spots (DBS) samples collected from non-human primates, carnivores, rodents, edentates, and ungulates by subsistence indigenous hunters as part of a wildlife conservation program, taking advantage of the discarded material from legal subsistence hunting, in the Yavarí-Mirin River basin (04°19′53″ S, 71°57′33″ W) in the Peruvian Amazon (Table S1). The sampling regions have a typical equatorial climate with an annual temperature of 22–36°C, a relative humidity of 80%–100%, and an annual precipitation of 1500–3000 mm. The project was first launched over a decade ago in 2007, to collect biological samples in the Amazon region with limited resources using a simplified DBS sampling strategy for wildlife pathogen surveillance. Subsistence hunters with an interest in participating in the research project were trained to perform blood collection from game animals. The consistent and simple collection methodology was applied prior to storage on different filter paper types. W3 or FTA paper was provided to the hunters. W3 was the most widely applied filter paper, particularly at the beginning of the project, due to the high cost or lack of availability of FTA cards. The main differences among the collected samples technically are storage methods, downwards storage time, and sample processing procedure.

In the field, the sampling activity was generally done between 1 and 2 h after the animals were hunted. After removing the viscera, the main blood vessels (cranial and caudal vena cava, and the thoracic aorta) were sectioned, and blood samples were collected accordingly. Approximately, 0.8 mL blood was spotted on either W3 or FTA storage paper depending on material distribution, followed by 2–3 h of ambient drying and storage in desiccants. DBS samples were kept in individual envelopes sealed in separate zipper closure bags at room temperature until sample transportation between 3 and 9 months (frequency being restricted by the remoteness of the study areas and the poor transportation infrastructure) (Table S1). Once in the laboratory, DBSs were immediately archived at −20°C. Additionally, whole blood samples (2–3 mL) from 44 local inhabitants were collected by venipuncture from voluntary participants, after providing written informed consent, and were transported in liquid nitrogen to the research lab to be stored at −80°C.

### 2.2 | Nucleic acid isolation

Nucleic acid was extracted from cold-chain stored human whole blood and wildlife DBS samples. Four nucleic acid extraction protocols were applied:

1. J_DNA: approximately 1 cm^2^ of storage paper was used and cut into small pieces with sterile scissors and diluted with 500 μL of PBS buffer. Diluted DBS discs were centrifuged at 6000 rpm for 2 × 20 s. After centrifugation, 200 μL supernatant was used. After adding 20 μL proteinase K and 200 μL Buffer AL in DNeasy Blood & Tissue Kit (Qiagen, Germany), the mixture was incubated at 56°C for 10 min. Downstream extraction was conducted following the manufacturer’s protocol. A volume of 50 μL per sample of human whole blood under sustained cold-chain storage was processed following the manufacturer’s protocol throughout the entire procedure;
2. J_R + D: scissor-sliced paper discs were diluted using 2 volumes (1120 μL) of AVL buffer with RNA carrier (Qiagen, Germany) and were centrifuged at 2000 rpm for 10 min. Nucleic acid was isolated from the supernatant with a QIAamp Viral RNA Mini Kit (Qiagen, Germany) according to the downstream manufacturer’s protocol. A volume of 50 μL human whole blood samples with sustained cold-chain storage was processed following the manufacturer’s protocol throughout the entire procedure;
3. G_D + R: filter papers were hole punched using a 3 mm hole punch cutter. Between 10 and 12 paper discs (0.70–0.85 cm^2^), roughly equivalent to 50 μL of whole blood, were used for nucleic acid isolation. DBS hole punches were diluted with 600 μL of RLT Plus Buffer and then were incubated at 55°C at 2000 rpm for 30 min in a ThermoMixer. Column-based DNA isolations were performed following Qiagen Quick-Start Protocol AllPrep DNA/RNA Mini Kit (Qiagen, Germany).
4. O_DNA: DNA was isolated from 2008 to 2018 with the QIAamp DNA Mini kit (Qiagen, Germany). Filter paper was hole punched using a 2 mm hole punch cutter. Between 25 and 30 paper discs (0.79–0.94 cm^2^) were diluted in Buffer ATL and proteinase K, followed by incubation at 56°C. The dilution was then used for the downstream column-based DNA purification following the manufacturer’s protocol. DNA extracts were stored over different periods of time (Table S1).

A total of 71 DNA extracts were processed by method one (J_DNA), 153 by method two (J_R + D), 15 by method three (G_D + R), and 36 by method four (O_DNA). Eighteen FTA DBS, three W3 DBS, and 30 human blood samples were used more than once for DNA extractions (Table S1).

Nucleic acid extracts were quantified and fragmentation analysed on an Agilent Tapestation 2200 system immediately after extraction, except DNA extracts from the O_DNA method which were stored at −20°C over different lengths of time prior to measurement. Fragmentation was assessed using the DNA Integrity Number (DIN) algorithm, in which the fluorescent signals of fragment size distribution determine integrity scores (a range of 1–10 values). The higher the value, the higher the nucleic acid integrity. Fragment size peaks were obtained from the nucleic acid migration images generated.

### 2.3 | Library preparation, hybridization capture and sequencing

Twelve nucleic acid extracts from four primate species with DIN ranging from 1.7 to 3.2 and fragment peaks from 433 to 7778 bp were used for downstream viral screening using hybridization capture (Table S2). All 12 samples were kept on FTA cards and were extracted using the scissor-sliced nucleic acid isolation method (described above as isolation method 2). To fully explore the potential existence of both DNA and RNA viruses, nucleic acid extracts were first reverse transcribed into double-stranded cDNA with a two-step cDNA synthesis using Invitrogen SuperScript^®^ Double-Stranded cDNA Synthesis Kit with SuperScript^™^ III Reverse Transcriptase and (Thermo Fisher Scientific, Waltham, Massachusetts, USA). Neither DNAase nor RNAase was applied to detect both RNA and DNA viruses. Illumina sequencing libraries were prepared from the double-stranded cDNA following the Meyer and Kircher (2010) protocol with slight modifications (Meyer & Kircher, 2010; Seeber et al., 2019). Briefly, reverse transcribed DNA was fragmented to 300 bp using an ultrasonicator in 50 μL volume (Covaris M220; Covaris, Woburn, MA, USA). Fragment size and quantity were assessed on an Agilent 2220 TapeStation with D1000 chips. End repair and adapter fill-in were performed on 42.5 μL sheared cDNA with the NEBNext kit (New England Biolabs, New England Biolabs, Ipswitch, MA, USA). Each sample was barcoded by adding a unique combination of 6-nucleotide tags with five amplification cycles and three technical replicates.

For hybridization capture reactions, a bait set (70-mer nucleotides) based on the fifth-generation Virochip (Yozwiak et al., 2012) was used to target a wide range of vertebrate viruses after excluding viral families with only invertebrates host, endogenous retroviruses, plant viruses, and bacteriophage. Three equimolar libraries were pooled for each reaction run. The reaction mixtures were incubated for 48 h at 60°C following the manufacturer’s instructions. After bead washing, the capture products were enriched using 15 cycles of on-bead PCR with P5/P7 bridge primers with KAPA HiFi HotStart ReadyMix (Roche, Basel, Switzerland). The enriched capture products were then cleaned using a MinElute PCR Purification Kit (Qiagen, Hilden, Germany), quantified on both Agilent 4150 TapeStation and Qubit system, and pooled at equimolarity. The final pool of enriched capture products was diluted to 8 pM with a Phix DNA control spike-in of 1% and sequenced on an Illumina Miseq platform (Miseq v2, 300 cycles, 2 × 150 bp paired-end reads; Illumina, San Diego, CA, USA).

### 2.4 | Bioinformatic pipelines

Demultiplexed raw reads were trimmed to remove adaptors with CUTADAPT v1.15 (Martin, 2011) and were filtered using a quality score above 20 and read length above 50 bp with a single window of 10 bp using TRIMMOMATIC v0.38 (Bolger et al., 2014). The retained paired-end reads were subsequently merged using FLASH v1.2.11 (Magoc & Salzberg, 2011) with a cut-off of 20 bp overlap. For comparison purposes, whole-genome sequencing data of four primate species were downloaded from European Nucleotide Archive (ENA) under Project PRJEB59576: Cacajao calvus (ERR10941572), Cebus albifrons (ERR10942010), Lagothrix lagotricha (ERR10941540) and Sapajus macrocephalus (ERR10941507). The four ENA datasets were subsampled to incrementally generate a set of data with 50,000, 100,000, 150,000, 200,000, 300,000, 450,000, 600,000, 750,000, 900,000, 1050,000, 1,200,000, 1,350,000 and 1500,000 reads by using seqtk (https://github.com/lh3/seqtk). The downloaded ENA data and corresponding subsampled data all went through the same quality control step as described above.

Direct mapping against mitochondrial genome references was performed using the pre-processed pair-end reads of the 12 non-human primate samples to identify mitogenome sequences with BWA (Li & Durbin, 2009). The bam files generated were processed to calculate the number of mapped reads, the coverage percentage, and the average coverage depth of mitogenome references using Samtools (Li et al., 2009) and bedtools (Quinlan, 2014). All reads that failed to map to the mitogenome references were retained using Samtools, which were then mapped against nuclear genomes of the corresponding species or closely related primate species with Bowtie2 (Langdon, 2015).

De novo mitochondrial genomes assembly was performed using the pair-end reads with Novoplasty (Dierckxsens et al., 2016), by taking the complete CO1 gene of corresponding species as a seed sequence. The mitochondrial genome of respective species or the most closely related species were downloaded from NCBI nucleotide database as assembly references. Assembled mitochondrial genome sequences were curated by mapping paired reads against them using BWA. Mapping results were sorted using Samtools and manually checked in Tablet (Milne et al., 2013) to generate a final consensus mitochondrial genome sequence. The total coverage of each mitochondrial genome assembly was calculated with bedtools. The seven complete mitochondrial genomes were annotated with MitoFinder (Allio et al., 2020).

For viral sequence detection, the 12 primate sequencing datasets were processed using two viral classification pipelines: (1) direct classification and (2) post-assembly classification. The direct classification was performed by using merged reads to search against the BLAST viral genome reference database with Blastn (−evalue 1e-5) to identify candidate viral sequences. The reads with viral hits were retained after removing all bacteriophage hits. The remaining candidates were aligned against the complete NCBI nucleotide database (−evalue 1e-10) to confirm that matched reads were more similar to viral sequences than non-viral ones. The candidate viral sequences were then filtered with the threshold of overall query coverage above 90%, identity above 85%, and bit-score greater than 190. The retained candidates were further curated by aligning candidate sequences to the corresponding virus genome. The reads identified as viral hits but with low complexity were removed due to high ambiguity. Post-assembly classification was performed by first removing host mitochondrial and nuclear genome sequences using Bowtie2 and Samtools, and rRNA using SortMeRNA (Kopylova et al., 2012). Retrained reads were de novo assembled using the Spades assembler (Bankevich et al., 2012). Generated contigs were taxonomically classified following two rounds of blast search with a minimum e-value of 1e-5 for searching against the viral reference database and 1e-10 for searching against the complete nucleotide database.

### 2.5 | Statistical analysis

To compare the storage capacity of two types of paper, plus cold chain storage and nucleic acid extraction efficiency of the four applied methods, a generalized linear mixed-effects model (GLMM) was fitted using the R package lme4 using a maximal likelihood (ML) method (Bates et al., 2015). The DIN (DNA integrity number) obtained from the Tapestation system was used as the response variable after being logged transformed. Five predictors were included as fixed effects: storage methods, extraction methods, logged interval days from collection in the field until being transported to the laboratory, logged interval days from storage in the laboratory to DNA extraction, and logged size of DNA fragment peak. Individual sample ID was taken as a random effect. The samples with zero-value in DIN or fragment peak variables were removed. In total, DNA quality results from 251 DNA extracts were used in the statistical analysis (72 from cold chain, 124 from FTA and 55 from W3). The fitted model was evaluated by using R package DHARMa with the simulateResiduals() function (Figure S1) (Hartig, 2022). We conducted anova tests using the R function anova() to evaluate the statistical significance of each fixed effect and using the rand() function to assess the random effect. Post hoc tests were performed to test the statistical differences of every two storage and extraction methods by using the R package emmeans (Searle et al., 2012) with Bonferroni method for p-value adjustments.

## 3 | RESULTS

### 3.1 | Storage and extraction methods effects on nucleic acid integrity

DNA integrity and maximal fragment size significantly deteriorated as storage time increased. No significant differences were detected as a result of the time lag between sample collection and frozen storage (Table 1). Storage and extraction methods both significantly affected the DNA quality retrieved (Table 1). In contrast, sample-specific effects (i.e. random effect from sample ID) did not significantly contribute to the overall variation of the fitted model (Table 1).

**FIGURE 1:**
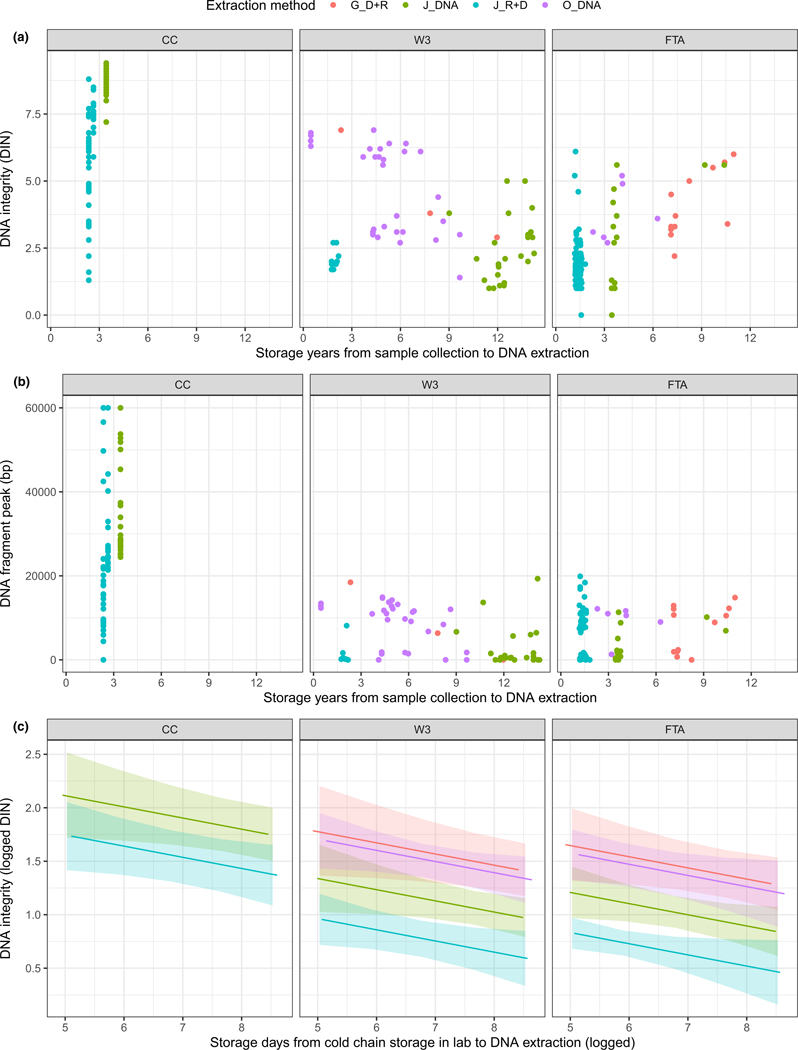
Plots from the isolated DNA and generalized linear mixed-effects models (GLMM). Panel (a) shows the DNA integrity (i.e. DIN value from Tapestation system) of each DNA isolate. Panel (b) shows the maximal DNA fragment retained in each DNA sample. Panel (c) shows the GLMM-based predictions of the DNA integrity (DIN) versus storage methods, extraction methods, and logged storage days from cold chain storage in the lab to DNA extraction. Types of filter paper used for sample storage: CC (cold chain storage), FTA (FTA card storage), W3 (Whatman filter paper no. 3). Nucleic acid extraction methods: J_DNA (extraction method 1: DNA-specific), J_R + D (extraction method 2: RNA-specific), G_D + R (extraction method 3: DNA and RNA combined), O_DNA (extraction method 4: DNA-specific).

**FIGURE 2:**
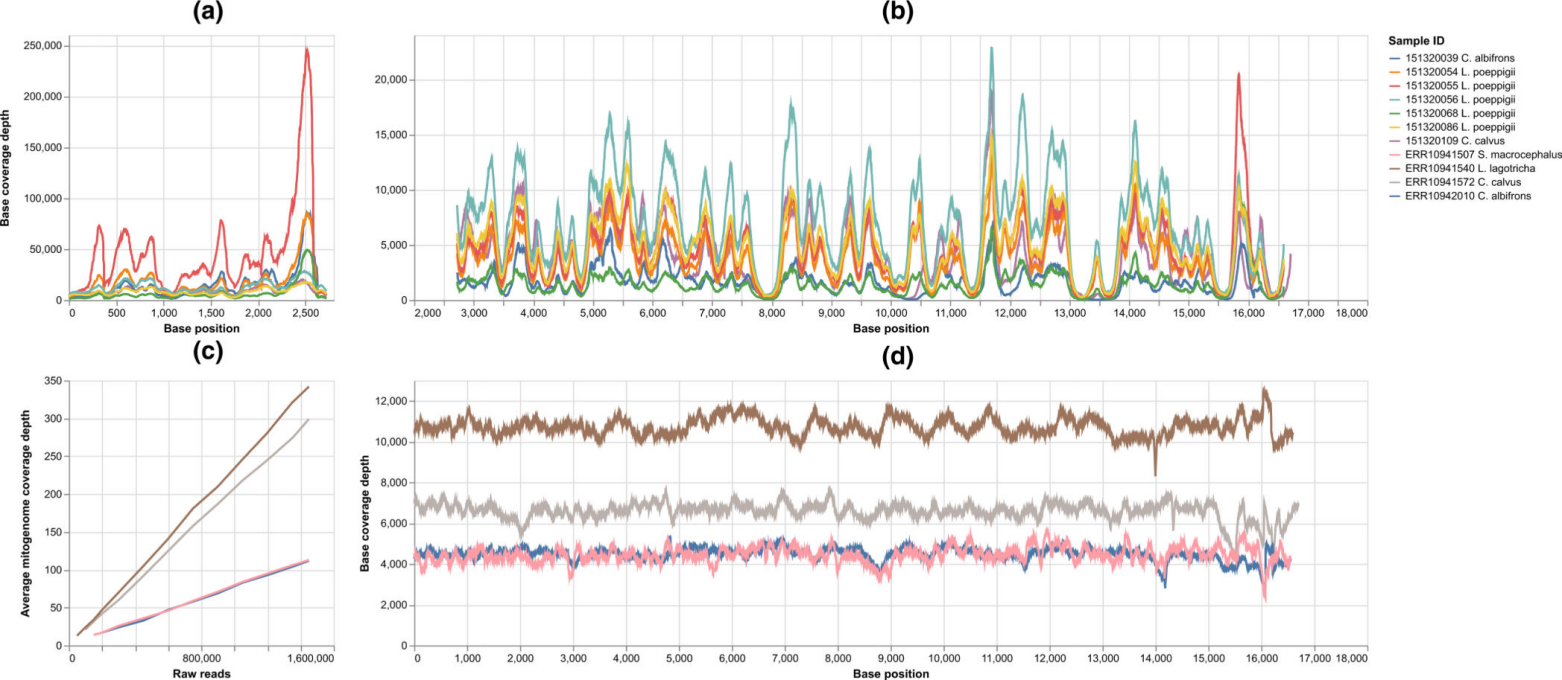
Results from the mapping against mitochondrial genome assemblies. Plot (a) shows the base coverage of seven mitochondrial genome assemblies that de novo assembled from target capture sequencing data from position zero to 2700. Plots (b) shows the base coverage of seven mitochondrial genome assemblies that de novo assembled from target capture sequencing data from position 2700 to the end position of each assembly. Plot (c) shows the average coverage depth of four mitochondrial genome assemblies that de novo assembled from downloaded ENA data versus the number of downsample sequencing reads. Plot (d) shows the base coverage of four mitochondrial genome assemblies that de novo assembled from whole-genome sequencing data downloaded from European Nucleotide Archive (ENA).

The comparison of storage methods showed that cold chain storage significantly improved the integrity of DNA retrieved (Figure 1c and Table 1). When compared with DBS samples of the same age or younger than cold-chain stored samples, the latter preserved longer DNA fragments (genomic DNA with fragment peaks above 15,000 bp) (Figure 1b). In contrast, no significant differences were detected between the two types of filter paper used (Table 1 and Figure 1c). Both FTA and W3 card stored samples yielded DNA fragments with maximal size of 20,000 bp (Figure 1b). DNA integrity (i.e. DIN) slightly increased for W3 card stored samples that were extracted within 6 years after collection (Figure 1a). For DBS samples stored longer than 9 years, DNA extracts had a DIN of ca 5.0 and maximal DNA fragment lengths below 10,000 bp (Figure 2a,b).

Three DNA-targeted extractions (method 1, 3 and 4) yielded significantly more intact DNA compared with that isolated using RNA-specific kit (Table 1 and Figure 1). Method 2 using a viral RNA-specific isolation kit yielded DNA with the lowest DNA integrity on average even for samples stored 1–2 years, regardless of which storage paper was used (Table 1, and Figures 1 and 2a). Significant differences were detected between J_DNA (method 1) and the other two DNA-specific extraction methods (method 3 and 4), but not between G_DNA (method 3) and O_DNA (method 4) (Table 1 and Figure 1c).

### 3.2 | Nuclear genome and mitochondrial genome retrieval

Although virus target capture was performed, there was a substantial non-targeted sequence generated per sample. The sequencing reaction generated 11.87 million reads for 12 primate samples with an average yield of 0.99 ± 0.35 million reads per sample, in which 11.24 million reads passed the quality control and could be merged into a single read (Table S3). The reads mapping to the host genome ranged from 0.64% to 51.21% of total reads from each sample, comprising 5479–684,464 reads with a median of 121,748 reads (Table S3). None of the 12 samples obtained more than 0.01% nuclear genome coverage. In contrast, 7.83%–61.88% of total reads were mapped to mitochondrial genome references. Six samples achieved 100% mitochondrial genome coverage, and two were below 50% coverage.

Seven complete and three partial mitochondrial genomes out of 12 collected samples for four primate species were de novo assembled: complete mitochondrial genomes for Cebus albifrons, Lagothrix lagothrica poeppigii, and Cacajao calvus; partial mitochondrial genomes for Sapajus macrocephalus (Table 2). A range of 120,805–659,177 reads were aligned to references in the de novo assembly pipeline, representing 17.05%–52.36% of the total sequencing data (Table 2). Mitochondrial genome assemblies ranged from 24× to 244,495× base coverage (Table 2). Non-uniform base coverage was observed for all seven mitochondrial genome assemblies. The mapping against the corresponding mitochondrial genome assemblies showed a slight increase in the number of aligned reads and average coverage depth compared with the results obtained from directly mapping against downloaded mitogenome references. The number of aligned reads in de novo assembly was correlated to the number of reads mapped to NCBI mitochondrial genome references (Table 2 and Figure S2a, *R*^2^ = 1, *p*-value < .0001). The coverage percentage of the respective references was positively correlated with the de novo assembly success (Table 2). In contrast, the indiscriminate whole-genome sequencing obtained significantly lower percentage of reads that were aligned to references, ranging from 0.43% to 1.31% depending upon species.

Compared with the unselective whole-genome sequencing data, the mitochondrial genome assembled from hybridization capture data obtained highly fluctuating coverage across the entire assemblies (Figure 2a,b). Coverage depth peaked at different gene coding regions across all three species (Figure 2a,b). Ribosome RNA regions had the highest coverage, especially for the end regions of the 16S rRNA gene (Figure 2a). By using subsampled whole-genome sequencing data, we observed that the sequencing effort required for successful de novo mitogenome assembly was highly related to the percentage of mitogenome sequences (Figure 2d). The two species with above 1.1% mitogenome sequence retrieval of the total sequencing effort required at least 100,000 reads to successfully de novo assemble complete mitochondrial genome with above 20× base coverage (Figure 2d and Table 2). The other two species with around 0.4% mitochondrial sequence required 300,000 reads to reach similar per base coverage (Figure 2d and Table 2).

### 3.3 | Virus classification

Between 2.38% and 24.34% of sequencing data with a median of 118,448 reads per sample aligned to viral references. The number of reads aligned to viral references was positively correlated to the number of reads aligned to mitochondrial genomes despite no statistical significance (Figure S2c, *R*^2^ = .21, *p*-value = .1305). Of those, 54.59%–93.47% of candidate viral reads were aligned to bacteriophage, ranging from 17,437 to 269,815 reads per sample (Table S4). After a second round of blast searches against the complete NCBI nucleotide database and stringent filtering, 24 reads from four samples were retained as potential candidate viral reads after removing bacteriophage reads. However, all 24 reads were low-complexity sequences and therefore cannot be confirmed as viral derived.

Post-sequence assembly classification results showed that a total of 1087 to 82,854 contigs were assembled from the nuclear genome and rRNA removed data. Ten out of 12 samples yielded 51 candidate viral contigs (Table S4). Four were retained after filtering out bacteriophage-like contigs but were also excluded as viral hits due to high ambiguity, low query coverage, and low sequence identity.

## 4 | DISCUSSION

### 4.1 | Nucleic acid storage and stabilization

In the present study, DNA integrity was strongly dependent on the extraction methods applied. High-temperature incubation protocols prior to nucleic acid isolation significantly improved the quality of DNA isolated from DBS preserved on both FTA cards and W3 paper. Kumar et al. (2019) reported that no differences were observed in DNA yields between two incubation-based isolation protocols consistent with our results (Kumar et al., 2019). In the process of DBS sampling, the DNA is absorbed, and captured within the porous and uniform matrix of the fibres once the biological fluid is applied to the cellulose-base paper. Under high-temperature incubation prior to isolation (generally applied in DNA-specific isolation), the physical nucleic acid bond to the fibre matrix is dissolved, which likely improves DNA dilution efficacy and DNA retrieval. In contrast, RNA-specific isolation kits can isolate DNA and RNA simultaneously but tend to result in DNA fragmentation, including for cold-chain stored samples.

DBS samples have been extensively applied for amplification-dependent analysis for a variety of research and diagnosis purposes (Hollegaard et al., 2009; Tuaillon et al., 2020; van Schalkwyk et al., 2019). More recently, whole-genome sequencing without selective amplification has been applied to DBS samples (Agrawal et al., 2021; Bassaganyas et al., 2018; Wu et al., 2019), with the results highly dependent on DNA fragmentation. A previous study demonstrates that relatively large DNA fragments (e.g. 1000 bp) can be preserved and amplified even for up to 11 years storage at ambient temperature in hospital settings of tropical regions, but amplification efficacy decreased when samples were stored longer than 9 years (Chaisomchit et al., 2005). PCR-based target detection performed differently on DNA isolated different preservation papers (Bezerra et al., 2021), in some cases with improved sensitivity when FTA cards were used (Hashimoto et al., 2019; Longhi et al., 2023; Tam et al., 2016). In the current study, the DNA integrity of DBS deteriorated significantly compared with that of cold-chain stored samples, but retained at a maximum of 20,000 bp fragments regardless of the storage period and filter paper used. Shorter storage periods within 3 years of collection preserved DNA fragments with an average length of 10,000 bp, even in some cases when a less optimal RNA-specific isolation method was applied. DBS samples stored longer than 9 years in a few cases preserved large DNA fragments, particularly for the samples preserved on FTA cards but the results were highly sample-dependent. This may be attributed to the chemical protection of the cards, which likely prevented large DNA fragments from enzymatic and environmental effects when storage was prolonged. However, one case of large-size DNA preservation (e.g. 20,000 bp DNA) was observed in DBS stored on W3 after nearly 15 years of storage. Therefore, minimal invasive DBS approaches regardless of filter paper used may hold great promise for genomic research on wildlife animals.

Collectively, our results suggest that DNA preservation of DBS on cellulose-based W3 was comparable to that of chemically-treated FTA cards under long-term storage conditions, with DNA retrieval efficiency strongly depending on the nucleic acid isolation methods applied. DNA fragmentation tends to deteriorate more seriously for samples stored on W3 over time, which may limit the application of the DBS samples in genomic-targeted research. Considering material expense and ease of implementation, DBS samples preserved on basic untreated filter paper could be an option for DNA-based projects.

When comparing the samples stored and extracted with the same methods, much of the observed degradation was stochastic and sample-specific, especially for FTA-stored samples. Blood samples were preserved in conditions of tropical temperature and humidity until they were transported to the laboratory every 6 months or annually. The lag time from sample collection to transportation should have introduced substantial among-sample preservation variation. However, our statistical results show that the in-between time did not significantly affect the overall DNA integrity. Even when the lag time was as short as 6 days, high degradation remained to be observed (Table S1). This suggests that the major DNA degradation most likely occurred at early stage when sampling was operated in tropical conditions, which all DBS samples in our study have suffered. Such factors may have contributed to the variable degradation profiles even though samples were subsequently stored under the same conditions at each sample site. Once properly stored on filter paper, DNA degradation continued but was slowed down significantly as indicated by the significant effect of the downward lag time between storage in the lab to DNA extraction. Besides, minor collection volume differences, microclimatic differences, or species-specific blood integrity may be responsible for the stochasticity but remain to be explored.

Overall, the sampling strategies with minimal invasiveness provide invaluable biological resources for wildlife research. Our sample collection method was integrated within a community-based participatory program aimed at improving the conservation and sustainability of natural resources and the livelihoods of local Amazon communities, who rely on subsistence hunting for food security (Jacob et al., 2023). The collection of blood samples from wild animals hunted for self-consumption by the local population allows for obtaining valuable biological material from wildlife in natural habitats without taking additional samples to those normally collected (Mayor et al., 2017). In our study area, the local population discards such biological samples. Therefore, the collection of biological samples is compatible with local culture; however, training is required to properly obtain, identify, and preserve the samples. This simple and low-cost method allows for the efficient collection of a high diversity of wild species hunted for self-consumption, allowing to improve the understanding direct health risks of local communities.

### 4.2 | Mining of capture sequencing data generated from degraded DNA

While metagenomic approaches enable rapid and comprehensive discovery and characterization of viruses, the approaches tend to be less suitable and less cost-efficient for highly degraded wildlife genetic material, particularly when the genome size of the study subject is large (McCartney-Melstad et al., 2016). Target capture sequencing can improve results from such samples as it enriches the yields of target sequences (Mamanova et al., 2010) and reduces background when applied to historical DNA (Willerslev & Cooper, 2005). Extensive studies have been conducted for evaluating the practicability of both DNA and RNA virus screening on DBS samples by using amplification-dependent methods (Cardona-Ospina et al., 2019; Lange et al., 2017; Smit et al., 2014). We targeted DNA and RNA viruses using a panel of RNA baits targeting a wide spectrum of vertebrate viruses. Our results showed that a median of ~15.94% of sequencing data per sample were viral candidate sequences based on blasting against the NCBI viral genome reference database, while ~83.39% of the candidates were bacteriophage hits. Neither mammalian RNA nor DNA viruses, after excluding bacteriophage reads, were identified using two distinct taxonomical workflows with stringent filtering steps. A previous large-cohort study in humans without any infectious disease indicated only a tiny fraction of ~5% reads in 42% of the study participants were viral reads (Moustafa et al., 2017). Among them, the number of RNA virus reads was particularly low (Moustafa et al., 2017). Viral titre in uncharacterized wildlife is unknown but can presumably be low in presumably healthy sampled wildlife. In addition, although RNA viruses can be amplified and detected after months of storage on FTA cards (Abdelwhab et al., 2011; Cardona-Ospina et al., 2019), RNA was likely to degrade more severely than DNA. The highly degraded nature of the DBS blood spots likely precluded the detection of RNA viruses. The absence of DNA viruses such as herpes or poxviruses (though positive for several by targeted PCR screening, data not shown) could suggest that none of the animals were infected, the titre was below detection limits, or the DNA was too degraded to detect them.

In target capture sequencing experiments, non-target sequences are commonly generated, especially when using broad capture panels or when capturing sequences with high similarity to unrelated genomic regions (Diroma et al., 2014; Picardi & Pesole, 2012). Previous studies targeting exome or nuclear ultra-conserved elements showed that mitochondrial sequences tend to be highly represented in the non-target sequences (Dashti et al., 2021; Diroma et al., 2014; Picardi & Pesole, 2012; Raposo do Amaral et al., 2015), consisting of ~1%–5% of reads in exome sequencing data (Samuels et al., 2013). A recent study demonstrates that DBS samples are robust for WGS analysis, allowing for the effective retrieval of whole mitochondrial genomes (Agrawal et al., 2021). In the current study, a median of ~43.67% of total viral-target sequencing data per sample were mitochondrial sequences. Sequences were heavily biased to the ribosomeal RNA regions consistently in all seven mitochondrial assemblies, particularly in the16S rRNA gene, leading to an average of ~10× higher coverage depth than the other regions. Hybridization capture allows for a high proportion of mismatched sites, which can improve the performance of detecting species that are highly divergent from those represented by reference sequences. Mitochondrial DNA experiences a slower degradation rate compared with nuclear DNA (Allentoft et al., 2012; Schwarz et al., 2009) likely due to the relative abundance of mitochondrial DNA compared with nuclear DNA (Bogenhagen & Clayton, 1978). We observed a positive correlation between the number of reads mapped to virus reference databases and those mapping to mitochondrial genome references though this did not reach statistical significance. This may indicate that viral baits with limited homology to mitochondrial sequences enriched them. Overall, our results demonstrated that non-target sequences generated in the sequencing of capturing non-host sequences could also be biologically valuable.

## 5 | CONCLUSION

Whatman filter paper no. 3 and FTA cards can be used for long-term DNA storage, with retrieval efficiency strongly depending on the nucleic acid isolation methods applied. DNA-specific isolation protocols with high-temperature incubation steps likely increase the efficiency of DNA retrieval from DBS samples. Overall DNA quality of DBS samples was significantly lower than that of cold-chain stored samples, while relatively large DNA fragments could still be preserved even after nearly 15 years of storage on basic filter paper without chemical protection. However, the application of DBS to viral detection with capture-based approaches, particularly for RNA viruses, was unsuccessful. Furthermore, off-target mitochondrial genome sequences could be effectively assembled from the target capture sequencing data, indicating the potential utility of DBS samples in wildlife genetic and genomic research.

## Supplementary Material

Table S1

Supplementary information

SUPPORTING INFORMATION

Additional supporting information can be found online in the Supporting Information section at the end of this article.

## Figures and Tables

**TABLE 1:** Results from generalized linear mixed-effects model that predicts the effects of sample properties and processing on DNA integrity.

Generalized linear mixed-effects model to assess DNA integrity (logged DIN)						
Fixed effects	Estimates (SE)	SE	df	*T* value	Chi2_LR	*p*-Value
(Intercept)	2.440	0.510	250.165	4.788		
Storage[FTA]	−0.908	0.179	151.737	−5.063	23.102	**<.0001** ***
Storage[W3]	−0.778	0.174	159.839	−4.469		
Extraction[J_DNA]	−0.446	0.120	249.587	−12.700	61.453	**<.0001** ***
Extraction[J_R + D]	−0.829	0.140	245.036	−5.924		
Extraction[O_DNA]	−0.094	0.143	245.718	−0.659		
log(Timelag.Collect_Cold)	−0.018	0.033	136.050	−0.545	0.296	.586
log(Timelag.Cold_Extract)	−0.104	0.053	250.566	−1.966	3.765	**.050** *
log(DNA_fragment_peak)	0.085	0.019	248.912	4.518	19.615	**<.0001** ***
Random effects					LRT	
Sample_ID (Intercept)	0.007	0.084			0.089	.764
Residual	0.120	0.347				
ICC	0.060					
Marginal *R*^2^/Conditional *R*^2^	0.724/0.740					
Post hoc test						
Storage method	**Contrast**	**Estimate**	**SE**	**df**	**T.ratio**	***p*-Value**
	CC-W3	0.908	0.185	194	4.908	**<.0001** ***
	CC-FTA	0.778	0.179	200	4.339	**.0001** ***
	W3-FTA	−0.130	0.870	257	−1.501	.404
Extraction method	**Contrast**	**Estimate**	**SE**	**df**	**T.ratio**	***p*-Value**
	G_D + R − J_DNA	0.446	0.123	259	3.628	**.002** *
	G_D + R − J_R + D	0.829	0.143	256	5.796	**<.0001** ***
	G_D + R − O_DNA	0.094	0.146	257	0.644	1.000
	J_DNA − J_R + D	0.383	0.081	144	4.740	**<.0001** ***
	J_DNA − O_DNA	−0.352	0.099	254	−3.551	**.003** *
	J_R + D − O_DNA	0.735	0.100	255	−7.323	**<.0001** ***


*Note*: Types of methods used for sample storage: CC (cold chain), FTA (FTA card), W3 (Whatman filter paper no. 3). Nucleic acid extraction methods: J_DNA (extraction method 1: DNA-specific), J_R + D (extraction method 2: RNA-specific), G_D + R (extraction method 3: DNA and RNA combined), O_DNA (extraction method 4: DNA-specific). Timelag.Collect_Cold: The lag time between sample collection to cold chain storage in lab. Timelag. Cold_Extract: The lag time between cold chain storage in lab to DNA extraction.

Bold denotes *p* values that have reached statistical significance.

Statistical significance:

* *p* values <.05

** <.01, and

*** <.001.

**TABLE 2:** Results from de novo assembled mitochondrial genomes and mapping against mitochondrial genome references.

		Mitogenome de novo assemble											
		De novo assemble with Novoplasty					Mapping reads to assemblies (BWA)			Mitogenome mapping to NCBI references (BWA)			
Species	Sample ID/ENA dataset ID	Assembled	Length (bp)	Aligned reads	Percentage of assembled reads	Average organelle coverage	Aligned reads	Percentage of mapped reads	Average coverage depth	Aligned reads	Percentage of mapped reads	Coverage percentage	NCBI accession of references
*Sapajus macrocephalus* (Large-headed capuchin)	151320002	Partial	16,541	370,679	29.37%	6768	457,509	35.77%	6651.2	460,123	34.42%	100.00%	NC_064167.1
	151320004	No								194,314	22.72%	42.40%	
	151320010	No								88,734	7.83%	35.90%	
	ERR10941507	Complete	16,544	424,724	0.44%	3879							
*Cebus albifrons* (White-fronted capuchin)	151320038	No								425,781	60.53%	87.00%	NC_002763.1
	151320039	Complete	16,552	203,081	41.79%	3705	287,005	58.15%	3904.63	277,246	53.28%	99.90%	
	ERR10942010	Complete	16,552	438,362	0.43%	3999							
*Lagothrix poeppigii* (Silvery woolly monkey)	151320054	Complete	16,594	342,405	37.85%	6232	439,258	46.38%	6014.44	435,214	44.09%	100.00%	NC_021951.1
	151320055	Complete	16,594	659,177	47.85%	11,997	805,386	57.04%	11,240.68	794,557	54.35%	100.00%	
	151320056	Complete	16,595	430,654	51.82%	7837	534,059	63.42%	8019.77	534,631	61.88%	100.00%	
	151320065	No								371,125	28.13%	95.10%	
	151320068	Complete	16,595	120,805	31.81%	2198	170,511	44.91%	2429.74	169,277	43.26%	100.00%	
	151320086	Complete	16,595	305,024	41.27%	5551	355,957	46.82%	5470.5	356,346	45.12%	100.00%	
*Lagothrix lagotricha*	ERR10941540	Complete	16,595	1,115,096	1.31%	10,146							
*Cacajao calvus* (uakari)	151320109	Complete	16,712	250,435	19.69%	49,311	351,124	26.92%	4977.01	349,481	25.83%	99.96%	NC_021967.1
	ERR10941572	Complete	16,700	702,212	1.17%	6349							


## Data Availability

The sequencing data generated in the current study are deposited in NCBI SRA under the BioPorject number PRJNA1024660. Seven de novo assembled mitochondrial genomes of three primate species were deposited in NCBI GenBank with accession numbers OR689233 (sample id: 151320109), OR689234 (sample id: 151320039), OR689235 (sample id: 151320054), OR689236 (sample id: 151320055), OR689237 (sample id: 151320056), OR689238 (sample id: 151320068), and OR689239 (sample id: 151320086).
