# Supplementary material for: Nucleic acid degradation after long-term dried blood spot storage
Source: Mol Ecol Resour. Author manuscript; Available in PMC 2025 Oct 6. (PMC12498251; doi:10.1111/1755-0998.13979)
Supplement: Supplementary information [file NIHMS2100888-supplement-Supplementary_information.docx]

Table S2. Information of samples used for target capture sequencing

| Species | Sample ID | Collected date(mm/dd/yyyy) | DNA isolation date(mm/dd/yyyy) | Storage days | DIN | Concentration (ng/µl) | DNA fragment peak/bp |
| --- | --- | --- | --- | --- | --- | --- | --- |
| *Sapajus macrocephalus* (Large-headed capuchin) | 151320002 | 09/15/2019 | 04/22/2021 | 585 | 2.1 | 11.4 | 475 |
|  | 151320004 | 10/16/2019 | 04/22/2021 | 554 | 2.6 | 86.9 | 1131 |
|  | 151320010 | 01/8/2020 | 04/29/2021 | 477 | 2.7 | 127 | 1323 |
| *Cebus albifrons* (White-fronted capuchin) | 151320038 | 10/27/2019 | 04/22/2021 | 543 | 3.8 | 47 | 1422 |
|  | 151320039 | 11/18/2019 | 04/22/2021 | 521 | 3.2 | 25.1 | 1695 |
| *Lagothrix poeppigii* (Silvery woolly monkey) | 151320054 | 09/05/2019 | 04/22/2021 | 595 | 1.9 | 18.7 | 433 |
|  | 151320055 | 09/25/2019 | 04/22/2021 | 575 | 1.7 | 11.9 | 451 |
|  | 151320056 | 09/28/2019 | 05/12/2021 | 592 | 2.2 | 43.5 | 898 |
|  | 151320065 | 01/31/2020 | 05/12/2021 | 467 | 2.3 | 21.5 | 444 |
|  | 151320068 | 02/01/2020 | 05/12/2021 | 466 | 1.7 | 35 | 482 |
|  | 151320086 | 2019 | 05/17/2021 | Not collected | 2.4 | 16 | 7778 |
| *Cacajao calvus* (Bald uakari) | 151320109 | 10/03/2019 | 04/22/2021 | 567 | 2.0 | 33.8 | 7747 |

Table S3. Samples used for target capture sequencing and the associated sequencing data.

| Species | Sample ID | Raw data | Percentage of capturable sequences per sample | Quality controled reads | Nuclear genome mapping | | | |
| --- | --- | --- | --- | --- | --- | --- | --- | --- |
|  |  |  |  |  | Alignment reads Bowtie2 | Percentage | Reference species | NCBI Accession |
| *Sapajus macrocephalus* (Large-headed capuchin) | 151320002 | 1355268 | 44.98% | 1336610 | 684464 | 52.21% | *Sapajus apella* | GCF_009761245.1 |
|  | 151320004 | 868893 | 27.97% | 855366 | 5479 | 0.64% |  |  |
|  | 151320010 | 1151788 | 41.37% | 1132646 | 10168 | 0.90% |  |  |
| *Cebus albifrons* (White-fronted capuchin) | 151320038 | 709778 | 25.49% | 703448 | 96799 | 13.76% | *Cebus albifrons* | GCA_023783575.1 |
|  | 151320039 | 526776 | 18.92% | 520339 | 161187 | 30.98% |  |  |
| *Lagothrix l. poeppigii* (Silvery woolly monkey) | 151320054 | 995900 | 31.67% | 987201 | 104014 | 10.54% | *Pithecia pithecia* | GCA_004026645.1 |
|  | 151320055 | 1475833 | 49.83% | 1461862 | 157486 | 10.77% |  |  |
|  | 151320056 | 871668 | 28.06% | 863916 | 139483 | 16.15% |  |  |
|  | 151320065 | 1347626 | 42.86% | 1319420 | 328930 | 24.93% |  |  |
|  | 151320068 | 395759 | 14.21% | 391272 | 91547 | 23.40% |  |  |
|  | 151320086 | 800604 | 25.46% | 789853 | 65671 | 8.31% |  |  |
| *Cacajao calvus* (Bald uakari) | 151320109 | 1365747 | 43.97% | 1352834 | 292611 | 21.63% |  |  |

Table S4. Results from two viral classification pipelines.

| Species | Sample ID | Meged reads | Direct blasting | | | | | Contig_blasting | |
| --- | --- | --- | --- | --- | --- | --- | --- | --- | --- |
|  |  |  | Total hits | Viral hits over total reads | Phage hits | Phage hits over total viral hits | Viral candidate reads against nt database | Assemble contigs | Viral candidate contigs against nt database |
| *Sapajus macrocephalus* | 151320002 | 1278954 | 56726 | 4.79% | 30967 | 54.59% |  | 2384 | 1 |
|  | 151320004 | 811355 | 139547 | 19.02% | 130384 | 93.43% | 15 | 37349 | 1 |
|  | 151320010 | 1071452 | 22704 | 2.38% | 17437 | 76.80% | 2 | 82854 | 2 |
| *Cebus albifrons* | 151320038 | 683402 | 152449 | 24.34% | 142609 | 93.55% |  | 6278 | 1 |
|  | 151320039 | 493602 | 97349 | 21.79% | 88707 | 91.12% |  | 1087 |  |
| *Lagothrix poeppigii* | 151320054 | 946998 | 166999 | 18.71% | 148564 | 88.96% |  | 8183 | 9 |
|  | 151320055 | 1411956 | 288654 | 22.07% | 269815 | 93.47% | 2 | 12974 | 1 |
|  | 151320056 | 842047 | 57511 | 7.72% | 41495 | 72.15% | 2 | 9171 | 1 |
|  | 151320065 | 1253901 | 142743 | 12.16% | 116317 | 81.49% |  | 15509 | 6 |
|  | 151320068 | 379661 | 49231 | 14.86% | 40878 | 83.03% | 1 | 7881 |  |
|  | 151320086 | 760330 | 63755 | 9.36% | 50204 | 78.75% | 1 | 17005 | 19 |
| *Cacajao calvus* | 151320109 | 1304223 | 200551 | 17.03% | 167970 | 83.75% | 1 | 6973 | 10 |


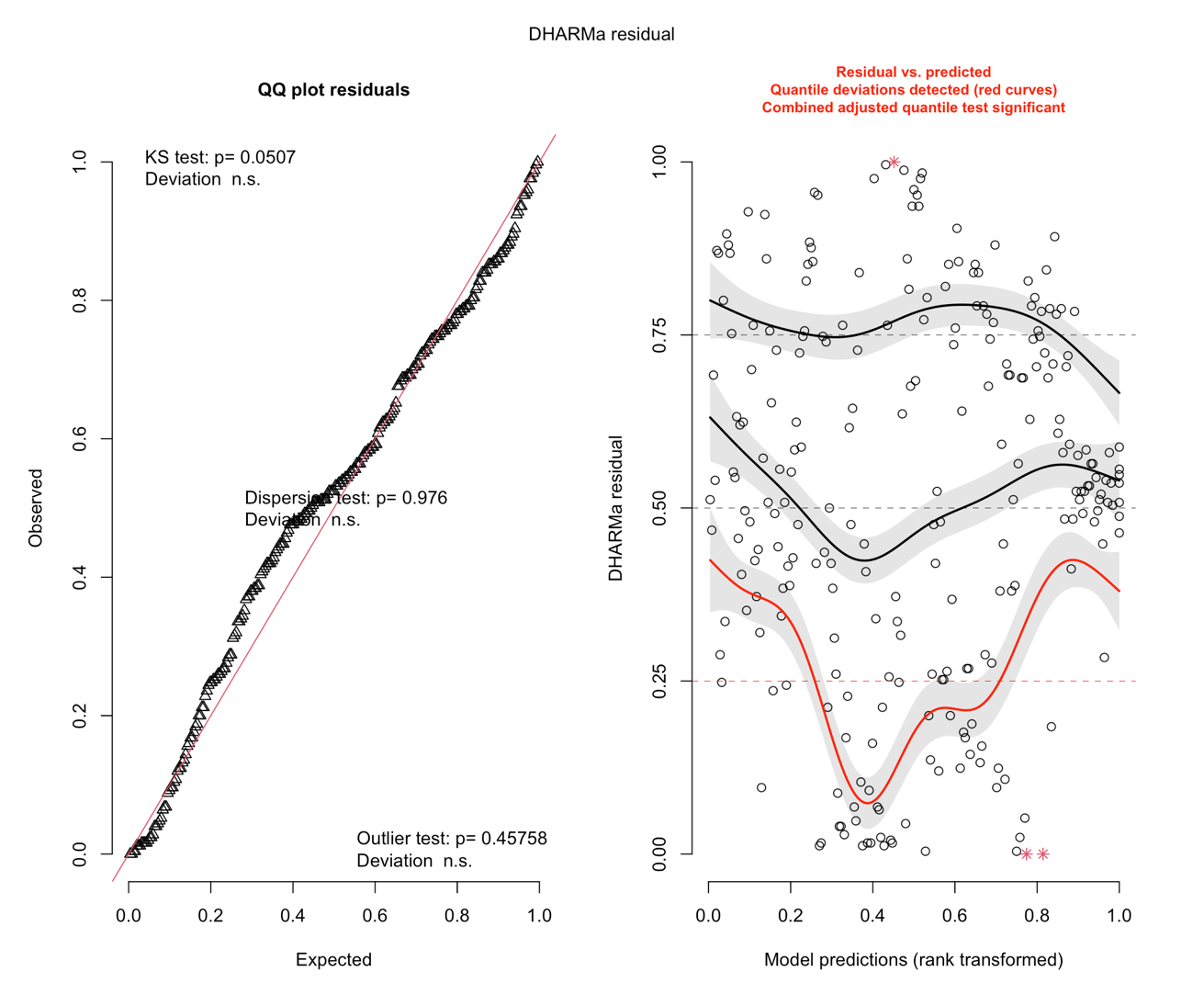


Figure S1. The panel of residual diagnostics plots for generalized linear mixed-effects models (GLMM) produced by the R package DARHMa. Note that the lack of significance denoted in the outputs from tests assessing the departure from modelling assumptions implies to accept the null hypotheses corresponding to a good fit of the model.


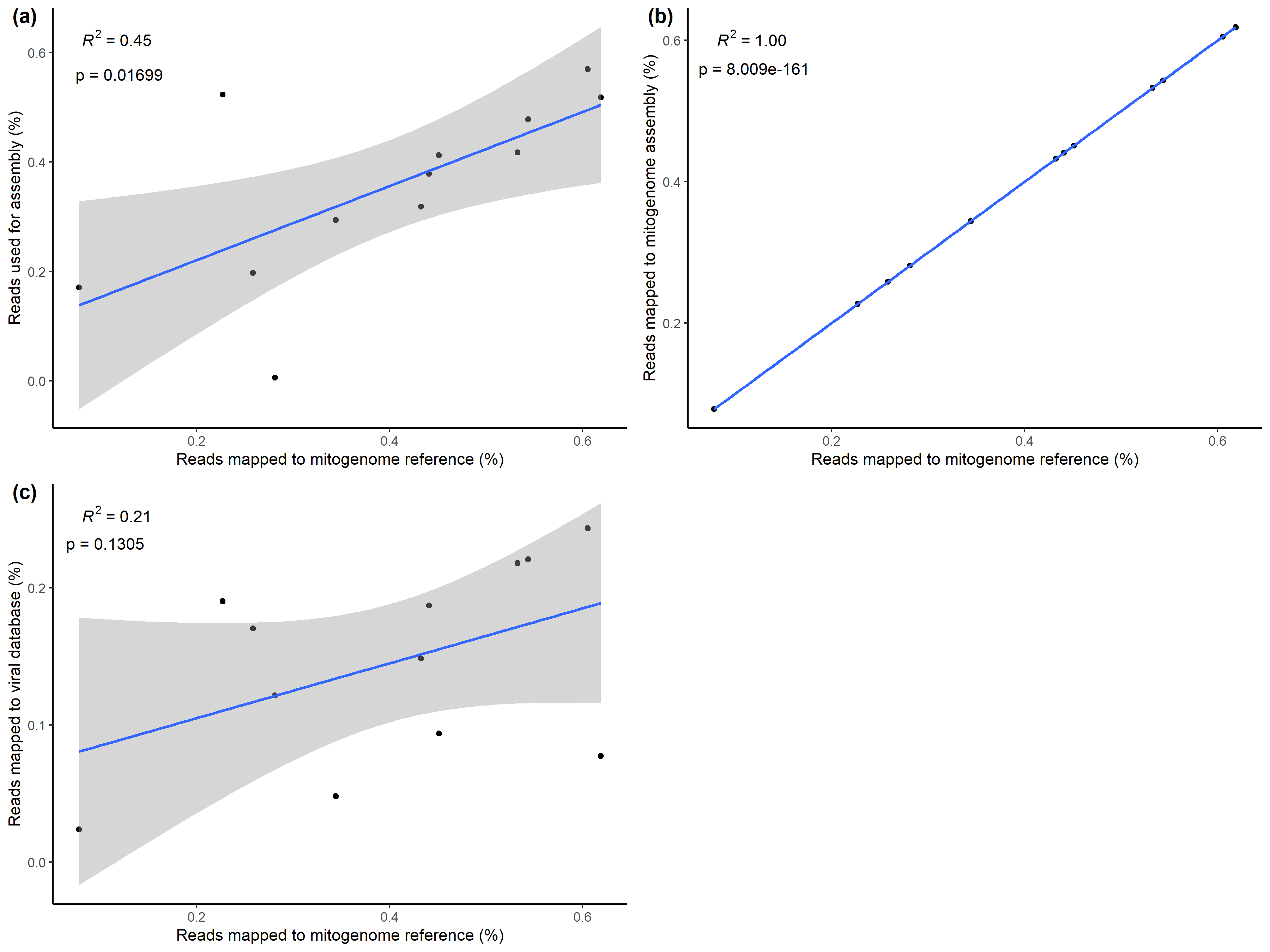


Figure S2. Plots from the linear models (LM) are shown. Plot (a) shows the percentage of reads mapped to mitochondrial genome references versus the percentage of reads used for de novo assemblies by Novoplasty. Plot (b) shows the percentage of reads mapped to mitochondrial genome references versus the percentage of reads mapped to de novo assembled mitochondrial genomes. Plot (c) the percentage of reads mapped to mitochondrial genome references versus the percentage of reads mapped to viral reference database.
